# Comparison of different ratios of propofol-ketamine admixture in rapid-sequence induction of anesthesia for emergency laparotomy: a randomized controlled trial

**DOI:** 10.1186/s12871-023-02292-w

**Published:** 2023-10-03

**Authors:** Mona Elsherbiny, Ahmed Hasanin, Sahar Kasem, Mohamed Abouzeid, Maha Mostafa, Ahmed Fouad, Yaser Abdelwahab

**Affiliations:** Anesthesia and Critical Care Medicine, Faculty of Medicine, Cairo University, Cairo, Egypt

**Keywords:** Emergency, Laparotomy, Rapid-sequence induction and intubation, Ketamine, Propofol, Hypotension

## Abstract

**Background:**

We aimed to compare the hemodynamic effect of two ratios of propofol and ketamine (ketofol), namely 1:1 and 1:3 ratios, in rapid-sequence induction of anesthesia for emergency laparotomy.

**Methods:**

This randomized controlled study included adult patients undergoing emergency laparotomy under general anesthesia. The patients were randomized to receive either ketofol ratio of 1:1 (n = 37) or ketofol ratio of 1:3 (n = 37). Hypotension (mean arterial pressure < 70 mmHg) was managed by 5-mcg norepinephrine. The primary outcome was total norepinephrine requirements during the postinduction period. Secondary outcomes included the incidence of postinduction hypotension, and the intubation condition (excellent, good, or poor).

**Results:**

Thirty-seven patients in the ketofol-1:1 and 35 patients in the ketofol 1:3 group were analyzed. The total norepinephrine requirement was less in the ketofol-1:1 group than in the ketofol-1:3 group, P-values: 0.043. The incidence of postinduction hypotension was less in the ketofol-1:1 group (4 [12%]) than in ketofol-1:3 group (12 [35%]), P-value 0.022. All the included patients had excellent intubation condition.

**Conclusion:**

In patients undergoing emergency laparotomy, the use of ketofol in 1:1 ratio for rapid-sequence induction of anesthesia was associated with less incidence of postinduction hypotension and vasopressor consumption in comparison to the 1:3 ratio with comparable intubation conditions.

**Clinical trial registration:**

NCT05166330. URL: https://clinicaltrials.gov/ct2/show/NCT05166330.

## Introduction

Anesthesia-induced hypotension is associated with serious organ failure and death [1]. The postinduction period constitute about one-third of intraoperative hypotensive episodes [2, 3]. Post-induction hypotension has several contributing factors; however, it is closely related to the anesthetic drugs [4]. Therefore, manipulation of induction agents makes post-induction hypotension likely preventable.

Patients undergoing emergency laparotomy are usually hemodynamically compromised and prone to post-induction hypotension; furthermore, these patients are usually at high risk of aspiration of gastric contents and require rapid-sequence induction of anesthesia and optimum intubating conditions.

Thus, induction of anesthesia for emergency laparotomy requires meticulous balance between achieving adequate hypnosis and maintenance of stable hemodynamics. Propofol is the commonest hypnotic agent worldwide. However, it is usually associated with hypotension especially in compromised patients. Ketamine produces dissociative anesthesia and sympathetic stimulation which provides more stable hemodynamic profile; however, ketamine is not widely used as a routine hypnotic [5].

Nevertheless, ketamine still has a role in induction of anesthesia in patients with shock and during procedural sedation [6, 7]. Ketamine is also used as analgesic adjuvant during general anesthesia [8].

Propofol/ketamine admixture (ketofol) was introduced in anesthetic practice aiming to counterbalance the side effects of the two drugs and to provide, consequently, the desired balance between adequate hypnosis and hemodynamic stability [9]. Ketofol is currently used with a diversity in the ratio between the two drugs which ranges between 1:1 and 1:10 [10–12]. Despite its frequent use in sedation and complete anesthesia, most of the available literature for comparisons of different ketofol mixtures was restricted to procedural sedation whose results are not applicable in induction of anesthesia due to the different desirable level of hypnosis and recovery. Therefore, the best combination of the two components of ketofol for induction of anesthesia is unknown.

The aim of this study is to compare two ratios of propofol and ketamine, namely 1:1 and 1:3 ratios, in rapid-sequence induction of anesthesia for emergency laparotomy regarding the vasopressor consumption, hemodynamic profile, adequacy of hypnosis and intubation conditions.

## Materials and methods

### Study design and order

This randomized controlled trial was conducted in Cairo University Hospital, emergency surgical theatre, from January to May 2022, after institutional Research Ethics Committee approval (October 10, 2021, No: MS-450-2021) and written informed consent was obtained from all subjects participating in the trial. The trial was registered prior to patient enrollment at clinicaltrials.gov (NCT05166330, Date of registration: 21/12/2021). All methods were carried out in accordance with the principles set forth in Helsinki.

### Population

Inclusion criteria included American society of anesthesiologist (ASA) physical status I-III patients aged 18–65 years old, scheduled for emergency laparotomy under general anesthesia.

Patients with a history of difficult intubation, abnormal airway examination, cardiac morbidities (impaired contractility with ejection fraction < 50%, heart block, arrhythmias, tight valvular lesions), patients on angiotensin converting enzyme inhibitors and angiotensin receptor blockers medications, patients with uncontrolled hypertension, patient with allergy of any of the study drugs were excluded from the study. Patients on vasopressor infusion, patients with high shock index (heart rate / systolic blood pressure > 1), body mass index > 35 kg/m2, increased intracranial tension and pregnant women were also excluded.

### Study protocol

Randomization was achieved by computer-generated sequence in a 1:1 ratio. Opaque sequentially numbered envelopes were prepared containing group assignment and drugs preparation instruction. The opening of the envelopes and drug preparation were done by an independent researcher with no further involvement in the study. The attending anesthetist and data collector were blinded to the study group.

### Drug preparation

Ketofol-1:1 group: 10 mL propofol (Propofol 1%, 10 mg/1 ml, FRESENIUS KABI DEUTSCHLAND GmbH, Deutschland) was mixed with 2 mL ketamine (Ketam 50 mg/mL, EPICO, Cairo, Egypt) and then diluted to a total volume of 20 mL to have a final concentration of 5 mg/mL propofol and 5 mg/mL ketamine.

Ketofol-1:3 group: 15 mL propofol (150 mg) was mixed with 1 mL ketamine (50 mg) and then diluted to a total volume of 20 mL to have a final concentration of 7.5 mg/mL propofol and 2.5 mg/mL ketamine.

Preoperatively, a trained anesthetist assessed the patients regarding the fasting hours, medical history, medications, laboratory investigation, as well as the patient’s airway.

In the operating room, electrocardiogram, pulse oximetry, and non-invasive blood pressure monitor were applied. After obtaining vascular access, slow intravenous 4 mg dexamethasone (DEXAMETHASONE 4 mg/mL – MUP, Medical Union Pharmaceutical, Cairo, Egypt) was given for prophylaxis against postoperative nausea and vomiting and for its analgesic property. In the supine position, baseline blood pressure was recorded as the average of three consecutive readings with difference < 10% in the systolic blood pressure.

Before induction of anesthesia for all study patients, volume assessment was done by measuring the baseline pulse pressure then giving a fluid challenge of 4ml/kg over 10 min. If the pulse pressure increased by > 15% of baseline, the patient was considered to be fluid responder, and the fluid challenge was repeated untill the increase in the pulse pressure was < 15% of baseline.

After 3-minutes preoxygenation, patients in the two groups received 1 mg/kg lidocaine in a separate syringe (Lidocaine Hydrochloride 2%, Sunny Pharmaceutical, Cairo, Egypt) plus 0.15–0.20 mL/kg of the prepared admixture until achieving clinical loss of consciousness (defined as no response to auditory command and the disappearance of a patient’s eyelash reflex).

After loss of consciousness, succinylcholine 1 mg/kg (Succinylcholine Chloride Injection 500 mg/5mL, Misr Co. for Pharm. Ind. S.A.E.) was administered over 5 s, and tracheal intubation was done through direct laryngoscopy after 60 s.

The intubation conditions were graded by the same anesthetist who performed intubation. The assessment included 1- ease of laryngoscopy (easy: jaw relaxed, no resistance to blade insertion; fair: jaw not fully relaxed, slight resistance to blade insertion; difficult: poor jaw relaxation, active resistance of the patient to laryngoscopy), 2- vocal cord position (easy: abducted; fair: intermediate/moving; difficult: closed), and 3- reaction to insertion of the tracheal tube and cuff inflation (Diaphragmatic movement/coughing) (easy: none; fair: one to two weak contractions or movement for less than 5 s; difficult: more than two contractions and/or movement for longer than 5 s).

The intubation condition was graded as excellent if all criteria are excellent, good if all criteria are either excellent or good, or poor if there was any criterion graded as poor [13].

When the trachea was intubated, mechanical ventilation was applied to obtain peripheral oxygen saturation > 95% and end-tidal CO_2_ between 30 and 40 mmHg and anesthesia were maintained by isoflurane in air/oxygen admixture (with target end tidal isoflurane 1%). Atracurium was administered after patient recovery from succinylcholine at a dose of 0.5 mg/Kg.

Any episode of hypotension (mean arterial pressure < 70 mmHg) was managed by a 5-mcg norepinephrine bolus (Norepinephrine 4 mg/4mL, Sunny Pharmaceutical, Cairo, Egypt), which was repeated if hypotension persists for 2 min).

Hypertension and tachycardia were defined as mean arterial pressure or heart rate > 120% of baseline, respectively. Persistent hypertension (blood pressure increasing after one measurement) was managed by intravenous 0.25 mg/kg propofol. Bradycardia (heart rate < 50 bpm) was managed by 0.5 mg of intravenous atropine.

After skin incision, hemodynamic and anesthetic management was according to the attending anesthetist discretion.

The primary outcome was total norepinephrine requirements during the period from induction of anesthesia until 16-minutes after intubation.

Secondary outcomes were incidence of post-induction hypotension, severe post-induction hypotension (mean arterial pressure < 60 mmHg), hypertension, bradycardia, and tachycardia during the period from induction of anesthesia until 16-minutes after intubation. Mean arterial pressure, heart rate was recorded at baseline, immediately after induction, after intubation, then every 2-minutes for 16-minutes after intubation. The Number of hypotensive episodes per patients, intubation condition (the number of patients with excellent, good, and poor intubation conditions), intubation time (time from insertion of the laryngoscope into the mouth until its removal after tracheal intubation), total ketofol volume, and total propofol and ketamine dose per weight.

Age, sex, weight, body mass index, American society of anesthesiologists-physical status, shock index, and preoperative fluid volume were also recorded.

### Statistical analysis

Sample size was calculated using the MedCalc Software version 14 (MedCalc Software bvba, Ostend, Belgium). In a pilot study on 14 patients (7 in each group), the mean norepinephrine dose in patients receiving ketofol 1:3 was 3.9 ± 5.2 mcg; and in patients receiving ketofol 1:1, norepinephrine dose was 0.7 ± 1.7 mcg. At alpha error of 0.05, we calculated that 68 patients would give 80% power to detect significant difference in the norepinephrine dose between the two groups. The number of prepared envelopes was 74 (37 envelopes per group) to compensate for possible dropouts.

Statistical package for social science (SPSS) software, version 26 for Microsoft Windows (Armonk, NY: IBM Corp) was used for data analysis. Categorical data were presented as frequency (%) and were analyzed by the Chi squared test. Continuous data were checked for normality using the Shapiro-Wilk test and were presented as mean ± standard deviation or median (quartiles) as appropriate. Continuous data were analyzed using the unpaired t test or the Mann Whitney test according to normality of the data. Repeated measured data were analyzed using the analysis of variance for repeated measures with post-hoc pairwise comparisons using the Boneferroni tests. A P-value less than 0.05 was considered statistically significant.

## Results

**Fig. 1:**
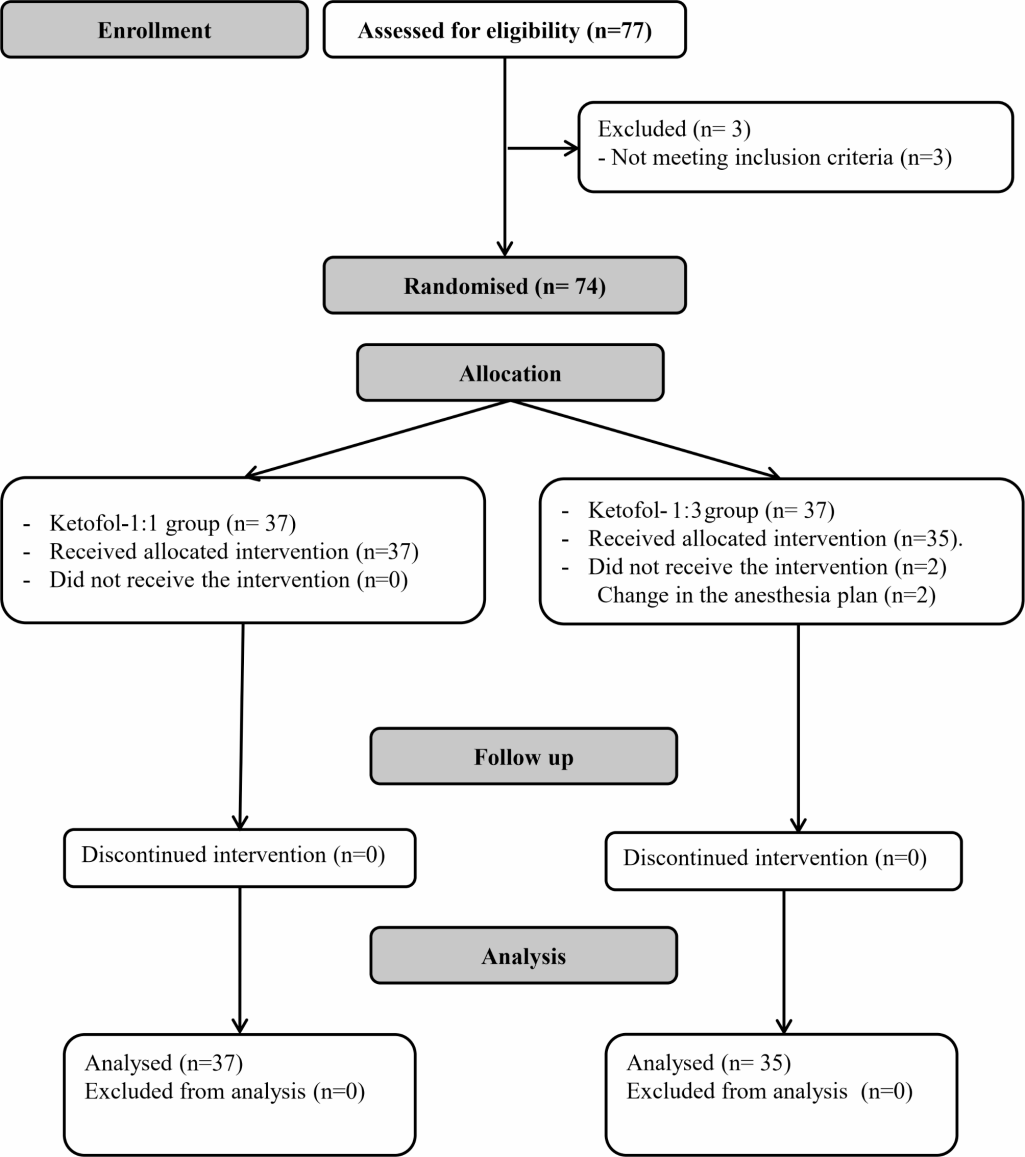
CONSORT’s flow chart

Seventy-seven patients were screened for eligibility, 3 patients were excluded for not fulfilling the inclusion criteria and 74 patients were equally randomized in to one of the study groups. Two patients in the ketofol 1:3 group did not receive the assigned intervention. Thirty-seven patients in the ketofol 1:1group and 35 patients in the ketofol 1:3 group were included and were available for the final analysis. (Fig. 1)

Patients’ demographic data and baseline hemodynamic data were comparable between the two groups. (Table 1)

**Table 1:** Demographic data and baseline hemodynamic characteristics. Data presented as mean ± standard deviation, median (quartiles), and frequency (%)

	Ketofol-1:1 group (n = 37)	Ketofol-1:3 group (n = 35)	*P*-value
Age (years)	41 ± 15	44 ± 16	0.416
Male sex	28 (76%)	23 (66%)	0.120
Weight (kg)	74 ± 13	73 ± 12	0.562
Body mass index (Kg.m^− 2^)	25 (22, 27)	26 (23, 29)	0.542
ASA-PS I II III	27 (73%) 9 (24%) 1 (3%)	25 (71%) 10 (29%) 0 (0%)	0.584
Comorbidities Diabetes Meletus Hypertension	7 (19%) 5 (14%)	8 (23%) 5 (14%)	0.681 0.925
Baseline heart rate (bpm)	87 ± 18	90 ± 14	0.899
Baseline mean arterial pressure (mmHg)	95 ± 9	99 ± 10	0.058
Shock index	0.66 ± 0.18	0.69 ± 0.13	0.500
Preoperative fluid (mL)	300 (245, 350)	300 (260, 480)	0.337
Intubation time (seconds)	40 (30, 40)	40 (30, 40)	0.834
Total ketofol volume (mL)	11.5 ± 3.9	10.5 ± 4.0	0.288
Propofol dose (mg/kg) median (quartiles) mean ± standard deviation	1.0 (0.5, 1.0) 0.8 ± 0.3	1.0 (0.8, 1.5) 1.1 ± 0.4	0.001
Ketamine dose (mg/kg) median (quartiles) mean ± standard deviation	1.0 (0.5, 1.0) 0.8 ± 0.3	0.3 (0.3, 0.5) 0.4 ± 0.1	< 0.001

ASA-PS: American Society of Anesthesiologists-Physical Status

The total volume of ketofol was comparable between the two groups. All the included patients had excellent intubation condition and the intubation time was comparable between the two groups. (Table 1)

The total norepinephrine requirement was less in the ketofol-1:1 group than in the ketofol-1:3 group, *P*-values: 0.043. The incidence of postinduction hypotension was less in the ketofol-1:1 group than in ketofol-1:3 group (6 [16%] and 13 [37%], respectively, P-value 0.044). Furthermore, the number of hypotensive episodes per patient were likely to be less in the ketofol-1:1 group than in the ketofol-1:3 group. (Table 2)

**Table 2:** Intraoperative hemodynamic outcomes. Data presented as median (quartiles), and frequency (%)

	Ketofol-1:1 group (n = 37)	Ketofol-1:3 group (n = 35)	*P*-value
Total norepinephrine requirement (mcg)	0 (0, 0)	0 (0, 5)	0.043
Incidence of hypotension	6 (16%)	13 (37%)	0.044
No. hypotensive episodes per patient	0 (0, 0)	0 (0, 1)	0.072
Incidence of hypertension	12 (33%)	9 (26%)	0.531
Incidence of tachycardia	12 (33%)	11 (31%)	0.864
Overall intubation condition Excellent Good Poor	37 (100%) 0 (0%) 0 (0%)	35 (100%) 0 (0%) 0 (0%)	1.000

The incidence of hypertension and tachycardia were comparable between the two groups and none of the included patients had severe hypotension nor bradycardia. (Table 2)

**Fig. 2:**
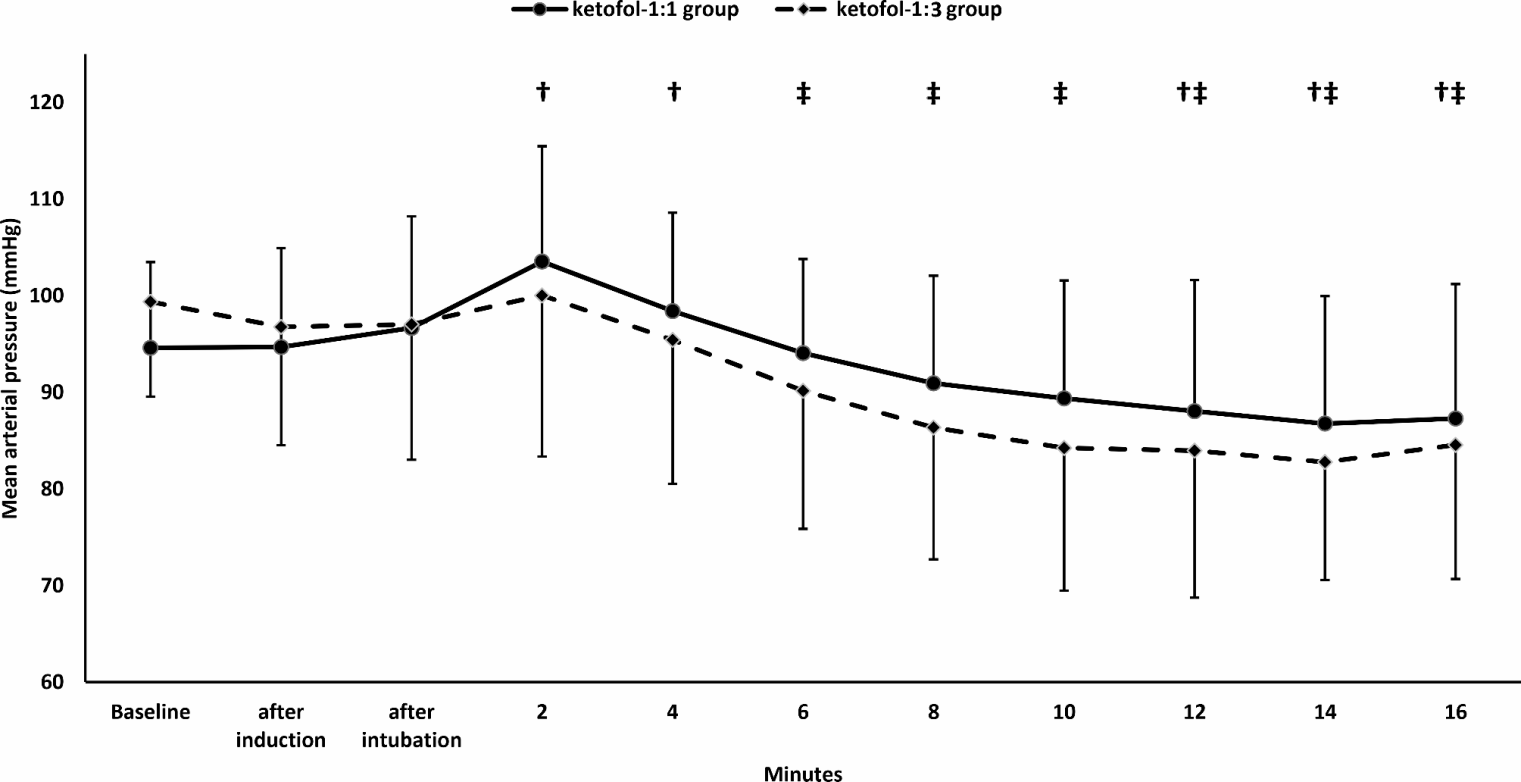
Mean arterial pressure. Markers are means and error bars are their standard deviations. † Denotes significance in relation to baseline value in the Ketofol-1:1 group, ‡ denotes significance in relation to baseline value in the Ketofol-1:3 group

**Fig. 3:**
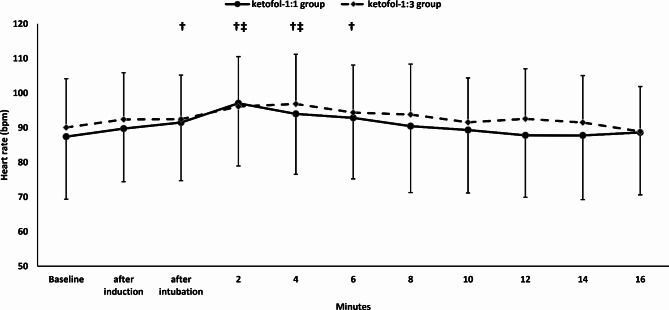
Heart rate. Markers are means and error bars are their standard deviations. † Denotes significance in relation to baseline value in the Ketofol-1:1 group, ‡ denotes significance in relation to baseline value in the Ketofol-1:3 group

The mean arterial pressure and heart rate were comparable between the two groups. (Figures 2 and 3)

The mean arterial pressure decreased in relation to the baseline value starting 12-minute postintubation in ketofol-1:1 group and 6-minute postintubation in ketofol-1:3 group. (Fig. 2)

The heart rate increased in both groups following the intubation. The heart rate became comparable to the baseline reading 8-minute and 6-minute postintubation in ketofol-1:1 and ketofol-1:3 group, respectively. (Fig. 3)

## Discussion

We compared two combinations of propofol and ketamine (1:1 and 1:3 ratio) for rapid-sequence induction of anesthesia in patients undergoing emergency laparotomy and found that the former dose (1:1) produced less incidence of hypotension compared to the 1:3 dose. The additive hypnotic action of the two drugs is well established [9]. Propofol is the most widely used hypnotic for induction of anesthesia and sedation due to its many favorable characteristics such as: rapid onset and offset without residual hang-over; antiemetic effect; and amnesia. However, its main disadvantage is the negative cardiovascular effect leading hypotension which is sometimes severe and serious [14]. Ketamine is another hypnotic agent which produces dissociative anesthesia and analgesia. Ketamine is characterized by a sympathomimetic effect which compensates the hemodynamic depressant effect of propofol [14, 15]. Thus, the combination of the two drugs provides a balance between the advantages and disadvantages of either drug alone [14].

Ketofol had been previously investigated in procedural sedation in the emergency department [16] and showed lower respiratory complication but conflicting results regarding its hemodynamic effect in comparison to propofol. Different ratios of ketofol were compared during procedural sedation and showed comparable hemodynamic effect [11]. When used for induction of anesthesia, ketofol showed better hemodynamic stability compared to propofol alone [17]. However, the hemodynamic effect of different ratios of ketofol for induction of anesthesia has not been yet explored. We hypothesized that at the induction dose, different ketofol ratios would have different hemodynamic effect. Our study is the first to compare different ratios of ketofol for induction of anesthesia in adult patients undergoing emergency laparotomy and showed superiority of the 1:1 ratio over 1:3 ratio. This finding differed from previous data in adults during procedural sedation; and pediatric population under total intravenous anesthesia [11, 12] that failed to find superiority for any ratio of the two drugs over the other. Our study included adult patients scheduled for emergency laparotomy which is usually associated with more hypotension than sedation due to the higher doses of hypnotic drugs as well as the effect of positive pressure ventilation; this might explain the of superiority of the 1:1 ratio in our results. Furthermore, our main objective was the hemodynamic profile of the two drugs while the objective of procedural sedation studies was the frequency of airway events.

In the current study, hypertension and tachycardia occurred in nearly 30% of patients in both groups. This could be due to either a hyperdynamic response to tracheal intubation or reactive hypertension due norepinephrine administration for hypotension treatment. We believe that Inadequate depth of anesthesia is unlikely to be the cause of this observation since the assigned drug was carefully titrated until reaching adequate hypnosis. We used clinical loss of consciousness as the hypnotic endpoint which is supported by the current evidence [18, 19] and all patients had excellent overall intubation condition. Using bispectral index in guiding induction of anesthesia is not feasible with the use of ketamine and lidocaine [20, 21]. During the maintenance period, appropriate depth of anesthesia was achieved by maintaining the end tidal isoflurane concentration at 1% [22]. In addition, the hyperdynamic response to tracheal intubation can occur despite the use of anesthetic doses of hypnotic drugs [23, 24].

The ketofol doses used in this study were within the range of what previously reported during induction of anesthesia [9, 10, 25]. We used lidocaine as an adjuvant which has an anesthetic-sparing effects and this helped in reaching adequate hypnosis using the current doses of ketamine and propofol [6, 25, 26].

Emergency gastrointestinal surgery is a high-risk surgery and is usually performed to control a life-threatening pathology [27]. Therefore, emergency laparotomy is commonly associated with major perioperative complications in 50% of the patients [28] and high mortality rates [29].

Hypotension is recognized as a major risk factor for perioperative morbidity and mortality [30] with the postinduction period being recognized as the most critical with a substantial proportion of the total hypotensive episodes during surgery [2]. Postinduction and pre-incision hypotension is associated with impaired cerebral perfusion [31] and postoperative kidney injury [2]. The most recognized threshold for perioperative hypotension that is related to postoperative morbidity and mortality was MAP 60–70 mmHg [32]. In this study we had the advantage of choosing a conservative threshold of MAP 70 mmHg as we used non-invasive blood pressure monitor which in turn tend to overestimate the low blood pressure values [33]. We used the absolute MAP value to define hypotension instead of relative reduction for several reasons; both absolute and relative hypotension threshold had similar postoperative morbidity risk [34]; the use of absolute MAP values is easier to the clinician; and the preoperative blood pressure does not reflect the patient’s ambulatory blood pressure [35].

Another advantage is the use of an opioid-free protocol for induction of anesthesia. Previous data showed that opioid-based protocol for induction of anesthesia increases the risk of postinduction hypotension and that lidocaine-based protocol provided stable postinduction hemodynamic with similar intubating condition in comparison to opioid-based protocol [25]. In addition, ketamine has good analgesic properties which could compensate for the absence of opioids.

We also had the advantage of including a special vulnerable group of (emergency surgery patients). Emergency surgery represents an independent risk factor for postinduction hypotension [3]; therefore, it is desirable to find the optimum anesthetic technique during these procedures to improve patient outcomes.

In this study, the period of assessment of postinduction hypotension was 16 min which is within the range of earlier studies assessing the postinduction hypotension (10–30 min) [3, 36–38]. Furthermore, longer assessment period would lead to unnecessary delay of an emergency surgery and shorter assessment period would not allow for proper hemodynamic assessment.

According to our findings, we suggest the use of ketofol 1:1 ratio would provide less incidence of hypotension during induction of anesthesia for emergency laparotomy, our study has some limitations such as being performed in a single center, excluding patients with major cardiac morbidities (e.g., stenotic valvular lesions and poor cardiac contractility) and including low number of patients with high ASA classification. Future studies will be needed to confirm our findings in other surgeries and other groups of patients. In this study, the blood pressure was monitored noninvasively as all our patients were hemodynamically stable and invasive blood pressure monitoring is not routine in such patients during induction of anesthesia. We did not record the postoperative course of the participants and their final outcomes since our main objective was the postinduction hypotension; therefore, future studies are needed to evaluate the effect of anesthetic choice on postoperative outcomes.

In patients undergoing emergency laparotomy, the use of ketofol in 1:1 ratio for rapid-sequence induction of anesthesia was associated with less incidence of postinduction hypotension and vasopressor consumption in comparison to the 1:3 ratio with comparable intubation conditions.

## Data Availability

The datasets used and/or analyzed during the current study are available from the corresponding author on reasonable request.
